# Assembling the Puzzle of Taxifolin Polymorphism

**DOI:** 10.3390/molecules25225437

**Published:** 2020-11-20

**Authors:** Roman P. Terekhov, Irina A. Selivanova, Nonna A. Tyukavkina, Igor R. Ilyasov, Anastasiya K. Zhevlakova, Alexander V. Dzuban, Anatoliy G. Bogdanov, Georgiy N. Davidovich, Gennadii V. Shylov, Andrey N. Utenishev, Dmitriy Yu. Kovalev, Anatoliy A. Fenin, Tatyana G. Kabluchko

**Affiliations:** 1 Department of Chemistry, Sechenov First Moscow State Medical University, Trubetskaya st. 8-2, 119991 Moscow, Russia; irinaselivanova@yandex.ru (I.A.S.); nonna_t@mail.ru (N.A.T.); igor@ilyasov.net (I.R.I.); azh-68@mail.ru (A.K.Z.); atom.66@mail.ru (A.N.U.); 2 Department of Chemistry, Lomonosov Moscow State University, Leninskiye Gory 1-3, 119991 Moscow, Russia; alex.dzuban@gmail.com; 3 Faculty of Biology, Lomonosov Moscow State University, Leninskiye Gory 1-32, 119991 Moscow, Russia; angeor@list.ru (A.G.B.); lembio@list.ru (G.N.D.); 4 Laboratory of Structural Chemistry, Institute of Problems of Chemical Physics, Russian Academy of Sciences, Acad. Semenov av. 1, 143432 Chernogolovka, Russia; genshil@icp.ac.ru; 5 Laboratory of X-ray Investigation, Merzhanov Institute of Structural Macrokinetics and Materials Science, Russian Academy of Sciences, Acad. Osipyan str. 8, 142432 Chernogolovka, Russia; kovalev@ism.ac.ru; 6 Institute of Materials for Modern Power Engineering and Nanotechnology, Mendeleev University of Chemical Technology of Russia, Miusskaya sq. 9, 125947 Moscow, Russia; fmkfenin@bk.ru; 7 Department of Technology, Ametis JSC, Naberezhnaya st. 68, 675000 Blagoveshchensk, Russia; tehnolog@ametis.ru

**Keywords:** taxifolin, flavonoids, active pharmaceutical ingredient, polymorphism, scanning electron microscopy, X-ray diffraction, variable-temperature powder X-ray diffraction, thermal analysis

## Abstract

A large amount of the current literature dedicated to solid states of active pharmaceutical ingredients (APIs) pays special attention to polymorphism of flavonoids. Taxifolin (also known as dihydroquercetin) is an example of a typical flavonoid. Some new forms of taxifolin have been reported previously, however it is still unclear whether they represent polymorphic modifications. In this paper, we tried to answer the question about the taxifolin polymorphism. Taxifolin microtubes and taxifolin microspheres were synthesized from raw taxifolin API using several methods of crystal engineering. All forms were described with the help of spectral methods, scanning electron microscopy (SEM), X-ray powder diffraction (XRPD), and thermal analysis (TA). SEM reveals that the morphology of the solid phase is very specific for each sample. Although XRPD patterns of raw taxifolin and microtubes look similar, their TA profiles differ significantly. At the same time, raw taxifolin and microspheres have nearly identical thermograms, while XRPD shows that the former is a crystalline and the latter is an amorphous substance. Only the use of complex analyses allowed us to put the puzzle together and to confirm the polymorphism of taxifolin. This article demonstrates that taxifolin microtubes are a pseudopolymorphic modification of raw taxifolin.

## 1. Introduction

Development of drugs is a long and expensive process. That is why pharmaceutical companies are poised to invest more money in research [1,2]. In such circumstances, crystal engineering provides an opportunity to reduce the cost of drug design and to modify biomedical properties of well-known compounds [3,4].

One of the primary goals of crystal engineering in drug design consists in obtaining the polymorphic landscape of the active pharmaceutical ingredients (APIs). Vernadsky defined polymorphism as a general property of materials [5]. This term has various definitions depending on the scientific field [6]. According to European Pharmacopeia, polymorphism is defined as follows: “the ability of a compound in the solid state to exist in different crystalline forms having the same chemical compound”. The U.S. FDA classifies amorphous, solvate, and hydrate forms as polymorphic. Polymorphism of APIs is an important subject in pharmaceutical science because the molecular packing may have a significant impact on physicochemical properties [7,8], biopharmaceutical parameters [9], and pharmacological activity [10].

A significant part of the current literature on polymorphism pays particular attention to flavonoids. From the point of view of chemical sciences, this group of compounds is a derivative of 1,3-diphenylpropane. They are secondary metabolites of plants [11]. Flavonoids are well known as bioactive compounds [12,13,14,15]. Implementation of these materials in medical practice is restricted by the limited water solubility and low bioavailability of flavonoids [16]. Researchers have attempted to modify the properties of these compounds via developing new polymorphic forms using crystal engineering [17].

**Figure 1:**
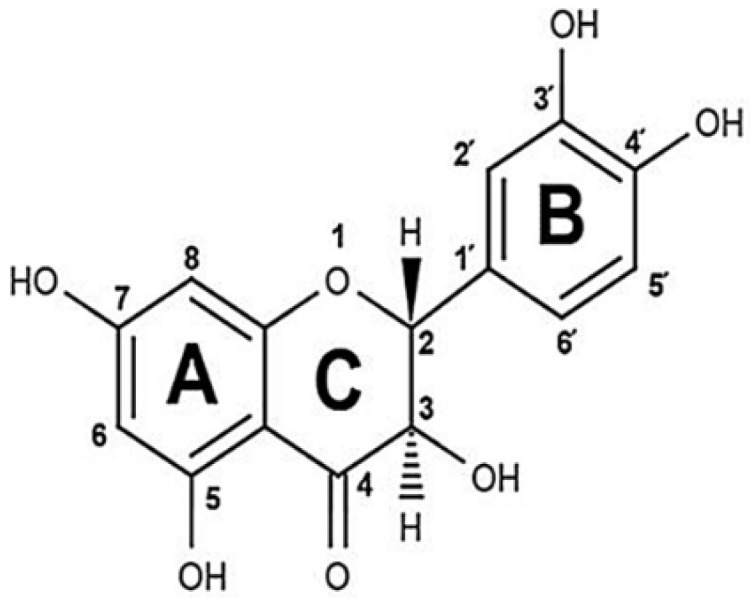
Chemical structure of taxifolin.

Taxifolin, also known as dihydroquercetin, is a commercially available flavonoid (Figure 1). Its main natural source is the butt-log portion of *Larix* spp. wood. Taxifolin has reducing properties and is well known as a food ingredient thanks to its antioxidant activity [18,19,20]. It is placed in the European Union market as a food supplement. This compound is also characterized by capillary-protective [21,22], neuroprotective [23,24], hepatoprotective [25,26], regenerative [27], antitumor [28], anti-inflammatory [29], antidiabetic [30], and antiviral activities [31,32]. Due to a wide range of pharmacological effects, this substance was registered as an API in Russia and it is being industrially produced by Ametis JSC.

In order to obtain a standard sample, a complex investigation of taxifolin was performed. There exist several articles that focus on the X-ray analysis of such samples, produced by a different manufacturer [33,34]. Physicochemical properties of taxifolin have previously been adjusted using chemical and physical modification [35,36], lyophilization [37], and nanodispersion formation [38]. There is still uncertainty, however, regarding whether different polymorphic forms of taxifolin exist.

This paper relays the analysis of different forms of taxifolin with the objective to try to categorize them as polymorphic modifications.

## 2. Results and Discussion

### 2.1. Morphology Analysis

**Figure 2:**
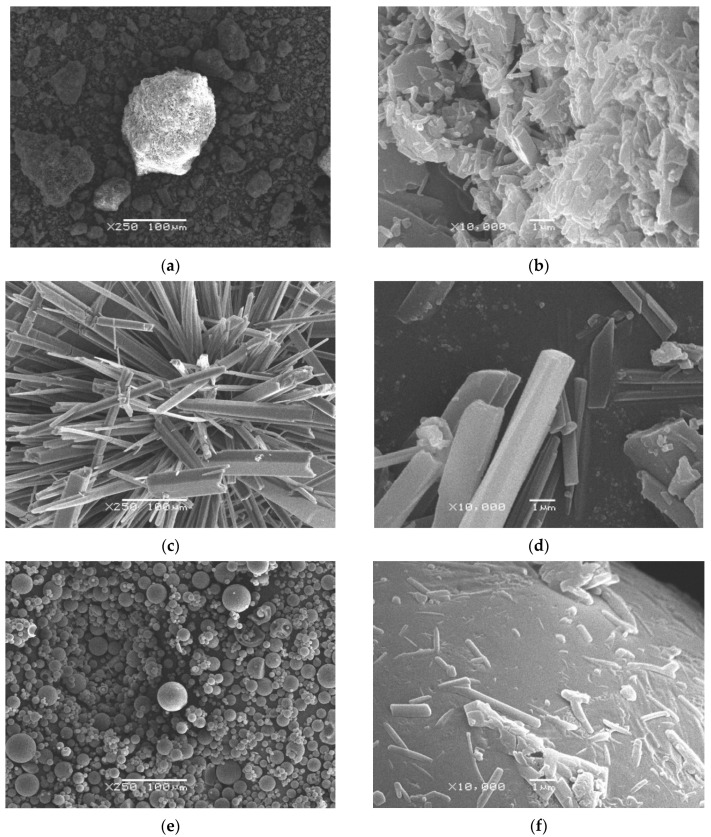
Photomicrography of different taxifolin forms: (**a**) raw taxifolin at 250× magnification; (**b**) raw taxifolin at 10,000× magnification; (**c**) taxifolin microtubes at 250× magnification; (**d**) taxifolin microtubes at 10,000× magnification; (**e**) taxifolin microspheres at 250× magnification; (**f**) taxifolin microspheres at 10,000× magnification.

For a detailed examination of the sample morphology, scanning electron microscopy (SEM) was performed. Microphotographs of different taxifolin forms are shown in Figure 2.

Figure 2a presents the morphology of raw taxifolin under 250× magnification. It is a fine powder with particles of an irregular shape. At the same time, under greater zoom (Figure 2b), we can see that the particles are not monolithic.

In contrast to this sample, taxifolin microtubes (Figure 2c,d) have a tubular structure. It is possible to see separate tubes or big, starlike crystals. The cross-section of microtubes is tetragonal or, in rare cases, hexagonal, while their surface is smooth with many longitudinal grooves.

The taxifolin microspheres, obtained by spray drying, are illustrated in Figure 2e,f at different magnifications. The presence of an internal cavity is confirmed by deformation of some particles (see Figure S1). Surprisingly, the morphology of the microsphere surface looks like an alloy of many particles (Figure 2f).

**Figure 3:**
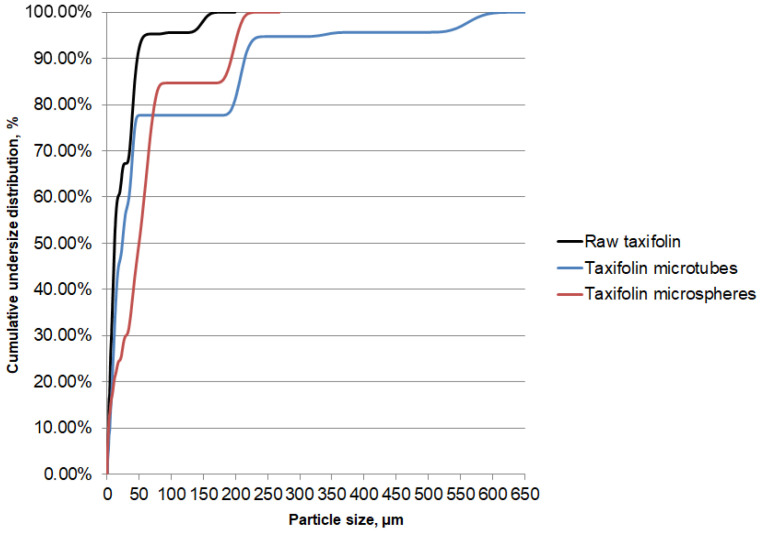
Cumulative undersize distribution of particles of different taxifolin forms.

The laser diffraction method was carried out to obtain an objective view of the particle size distribution (Figure 3). It was found that raw taxifolin had the smallest particles compared to other samples. Its median particle size was 11.73 µm, while the majority of particles did not exceed 46.90 µm. The largest median value 49.00 µm was found for taxifolin microspheres. In general, taxifolin microtubes demonstrated the biggest particles: *X*_10_, *X*_50_, and *X*_90_ were 4.18, 23.80, and 214.00 µm, respectively (Table 1).

According to morphology analysis, all samples are characterized by different shapes and sizes. It was important in our study to preserve taxifolin’s molecular structure in all samples, so we used a complex of spectral methods to confirm it.

### 2.2. Spectral Analysis

A taxifolin molecule contains two conjugated systems (ring A and ring B), which are chromophores. UV spectroscopy may be used to confirm the absence of structural degradations during the processing. UV spectra of taxifolin solutions, obtained from different modifications, show an absorption maximum at *λ* = 228.31 ± 0.27 nm (see Figure S2).

Mass spectra of taxifolin samples are characterized by the presence of peaks with the following *m*/*z*: 303, 417, and 607 (see Figure S3). The first represents the peak of quasi-molecular ions. Apparently, the last corresponds to the peak of the taxifolin dimer obtained through electrospray ionization, therefore the existence of all obtained signals may be explained by the structure of taxifolin.

NMR ^1^H spectra of different taxifolin forms look similar (see Figure S4). Interpretation of the signals is shown in Table S1.

Taxifolin’s structure was thus confirmed. To obtain more information about the nature of taxifolin forms, we continued our investigation using thermal analysis.

### 2.3. Thermal Analysis

**Figure 4:**
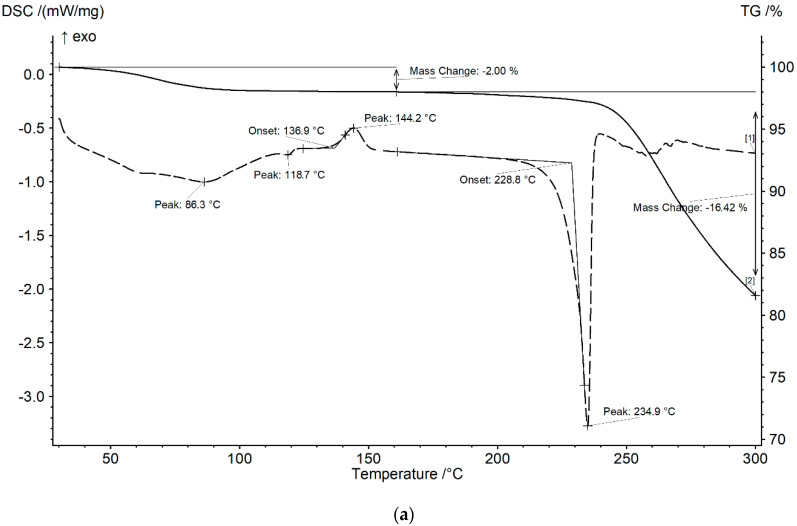

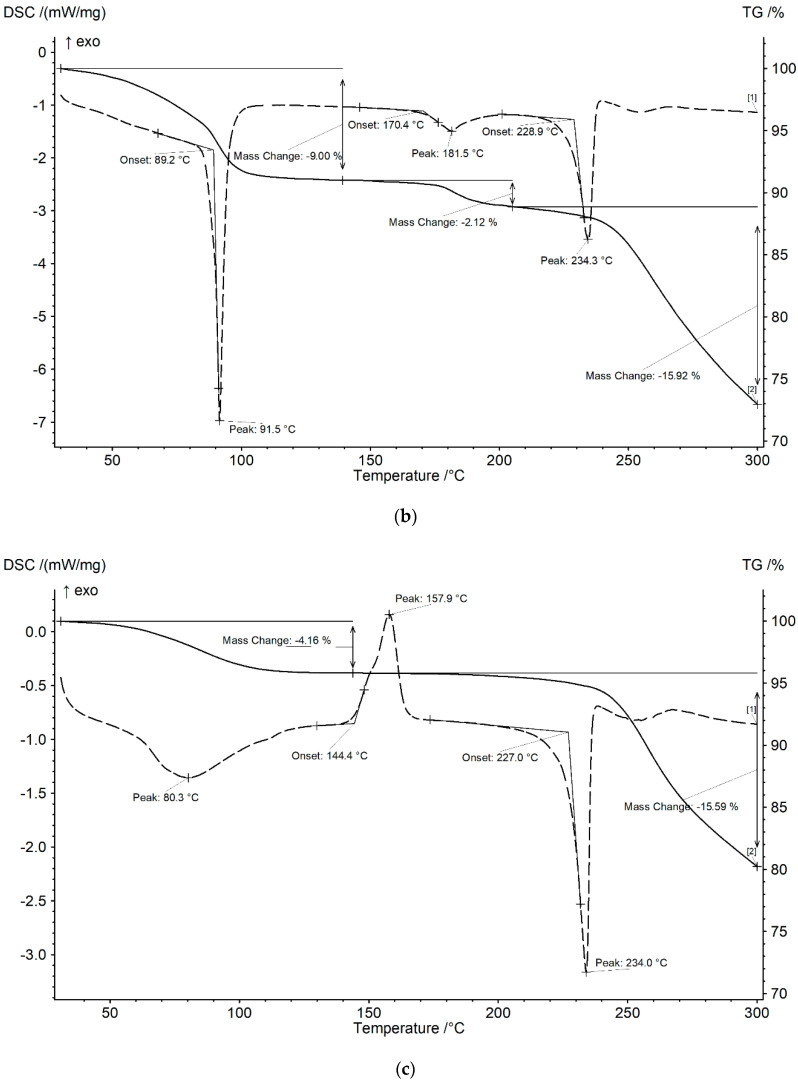
Thermograms of different taxifolin forms: (**a**) raw taxifolin; (**b**) taxifolin microtubes; (**c**) taxifolin microspheres.

Thermal analysis is one of the most convenient and informative methods that can be applied to study API polymorphism. Differential scanning calorimetry (DSC) and thermal gravimetric analysis (TG) of raw taxifolin, taxifolin microspheres, and taxifolin microtubes are presented in Figure 4.

The melting point of taxifolin samples lies at 228 ± 1 °C, and the process is accompanied by decomposition, suggesting that all modifications eventually transform into high melting forms (see Figure S5). There are, however, essential differences in preceding thermal profiles. DSC curves of raw taxifolin (Figure 4a) and microspheres (Figure 4c) exhibit exothermic effects at ca. 137 and 144 °C respectively, which can be interpreted as cold crystallization of an amorphous structure to a crystalline one.

Apparently, all samples contain moisture, physically sorbed water, which is completely released at 120 °C. Moreover, the DSC of taxifolin microtubes shows a pronounced endothermic effect at ca. 89 °C, most likely indicating the elimination of crystal solvent (Figure 4b). Simultaneous thermal analysis-mass spectroscopy (STA-MS) shows it is water (see Figure S6), which is fully consistent with our previous X-ray crystallography data [36]. Hence, this transformation should be considered as a transition from a hydrate to an anhydrous form. TG data suggest, given the superposition of mass loss steps, that taxifolin microtubes are a hydrate form of taxifolin.

For taxifolin microtubes, there is an endothermic effect starting from ca. 170 °C. This peak can be attributed to decomposition of urea contained in taxifolin tubes. This hypothesis is supported by STA-MS (see Figure S6): characteristic ions’ [39] intensity peak maxima coincide with the mass loss step—*m*/*z* 14 (N^+^), 15 (NH^+^, CH_3_^+^), 16 (NH_2_^+^), 17 (NH_3_^+^, OH^+^), 28 (CO^+^, N_2_^+^), 29 (HCO^+^), 42 (NCO^+^), 43 (HNCO^+^), 44 (CO_2_^+^). Even though the amount of urea molecules was small, it could be considered as an inclusion either in microtubes’ cavity or in the crystal structure. Thus, X-ray powder diffraction (XRPD) was performed to elucidate it.

### 2.4. X-ray Analysis

**Figure 5:**
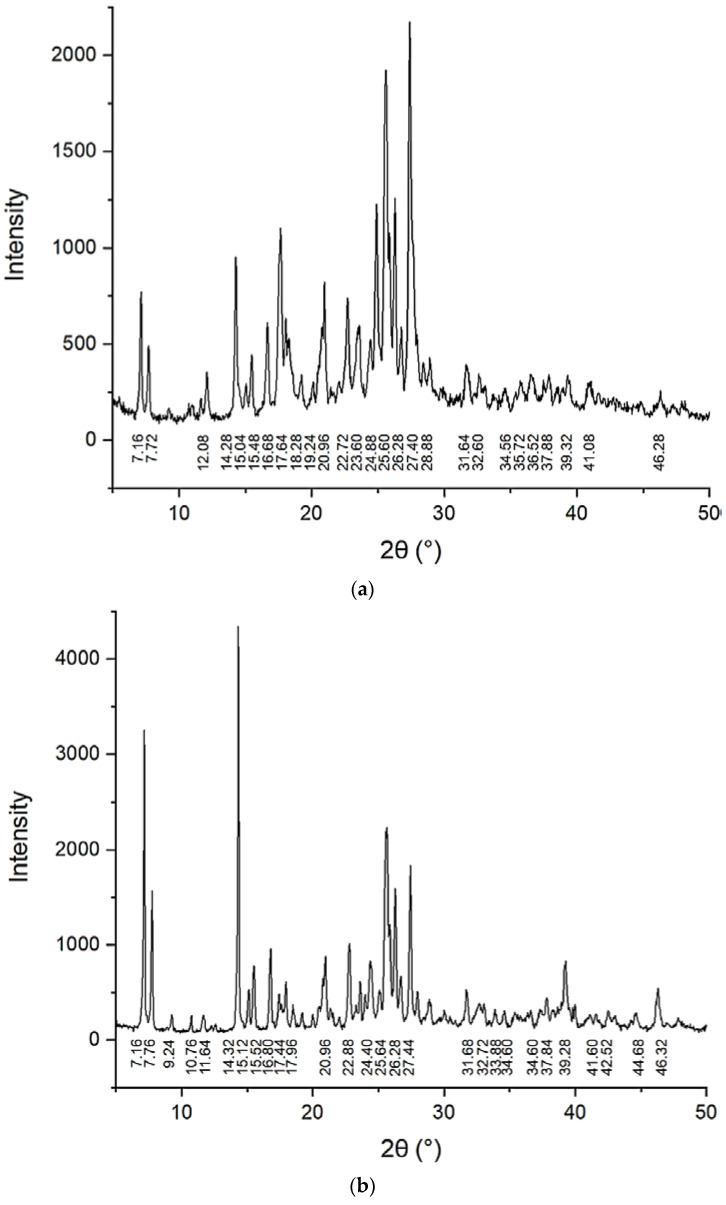

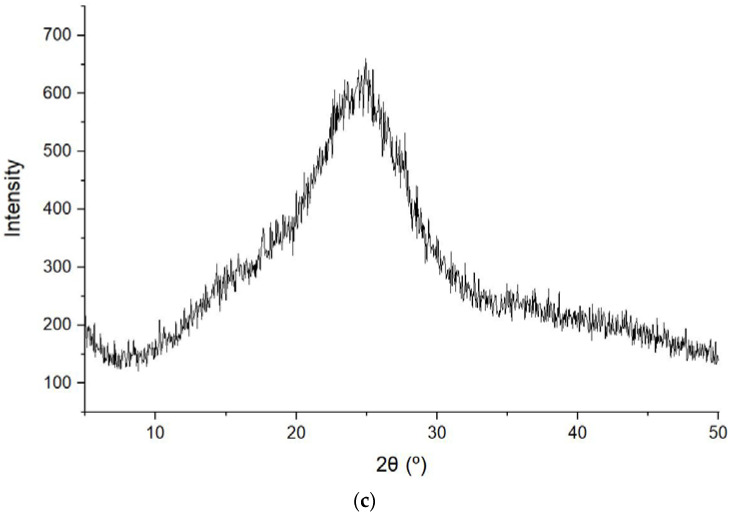
X-ray powder diffraction (XRPD) of different taxifolin forms at room temperature: (**a**) raw taxifolin; (**b**) taxifolin microtubes; (**c**) taxifolin microspheres.

XRPD is one of the most popular methods for the analysis of polymorphic modifications. All taxifolin forms studied in this paper have different XRPD patterns (Figure 5). The XRPD pattern of raw taxifolin is characterized by peaks at 2*θ* 7.16, 7.72, 14.28, 15.04, 15.48, 17.64, 20.96, 24.88, 25.60, 26.28, 27.40, 31.68, 34.56, 37.88, 39.32, and 46.28 (Figure 5a). Taxifolin microtubes have a similar XRPD pattern when compared to raw taxifolin, but nevertheless are not identical (Figure 5b). Additionally, a previous article reported a so-called taxifolin form I, which has a similar XRPD pattern but is characterized by another morphology—it was described as blocks [40]. The XRPD pattern of microspheres is characterized by an amorphous halo (Figure 5c).

Upon closer inspection, XRPD data exhibit strong differences in the intensity of diffraction peaks. The XRPD pattern of the raw taxifolin includes a higher number of diffraction peaks. The XRPD pattern of the microtubes shows some new peaks with low intensity at 2*θ* 9.24, 10.76, 11.64, 33.88, 42.52, and 44.68. These differences between XRPD patterns may correlate with changes in the crystal structure.

A theoretical calculation of urea XRPD patterns based upon X-ray crystallography data published previously [41] showed that crystals of taxifolin microtubes contain no free urea. This hypothesis is suggested by the absence of peaks at 001 (2*θ* = 28.2°), 110 (2*θ* = 33.8°), and 111 (2*θ* = 44.5°). Furthermore, with the help of the MOLSV program, it was ascertained that the van der Waals volumes of urea and water molecules were 40.4 Å^3^ and 13.1 Å^3^ respectively. According to taxifolin structural data, available in the Cambridge Structural Database (CCDC) [42] under identifier LORKEI02, a channel exists in the direction 010. The diameter of these channels is 45 Å. The urea probably forms part of the taxifolin solid phase acting as a guest molecule that does not take part in crystal structure formation, and it is most likely amorphous.

**Figure 6:**
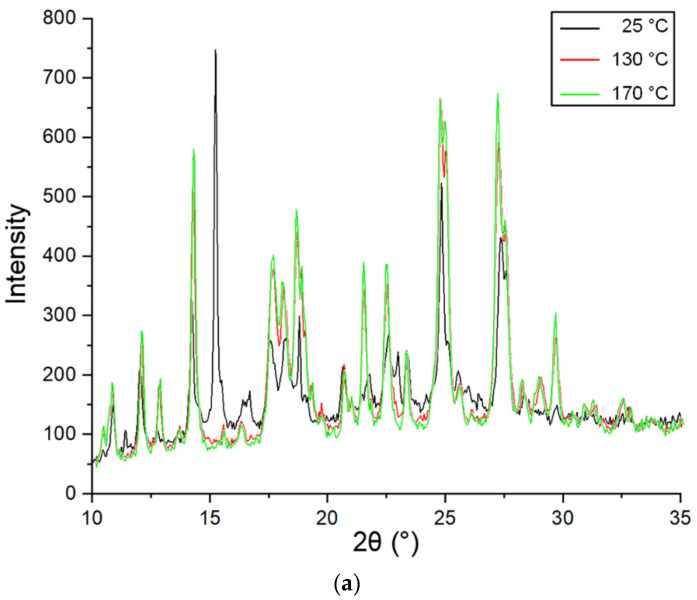

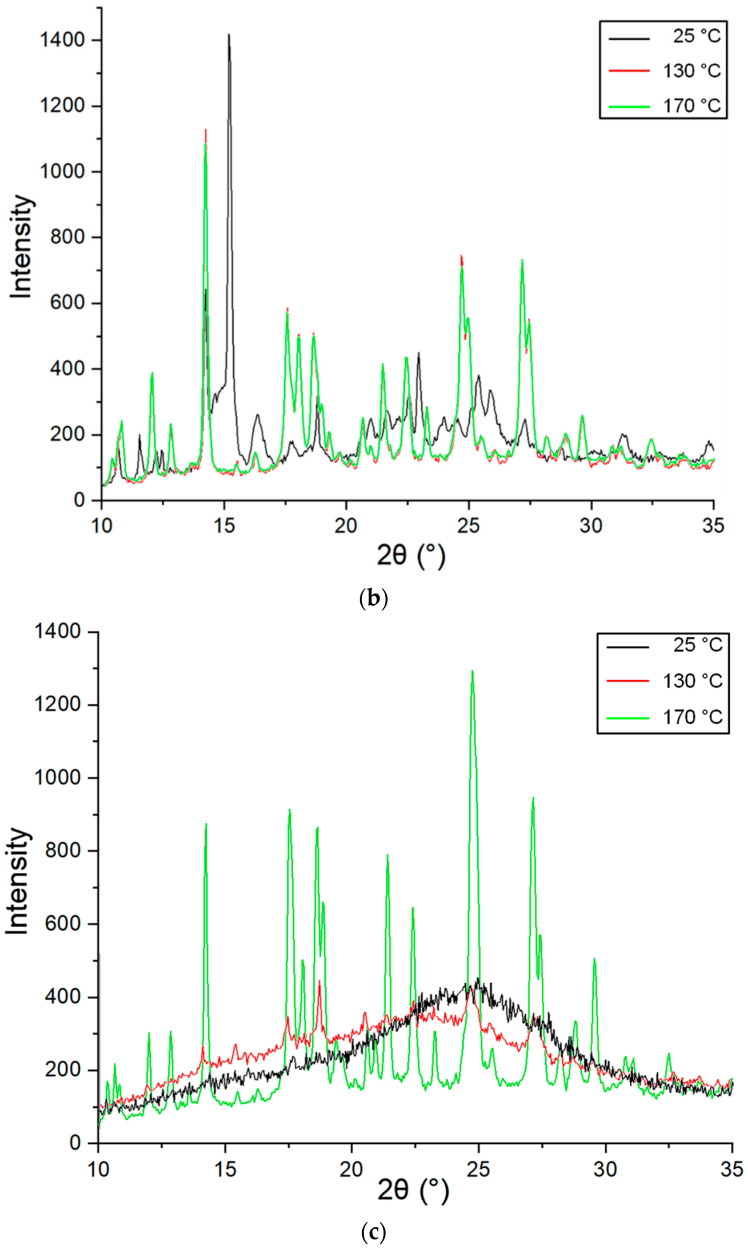
In situ variable-temperature XRPD patterns for different taxifolin forms: (**a**) raw taxifolin; (**b**) taxifolin microtubes; (**c**) taxifolin microspheres.

To clarify the nature of the phase transition, we conducted in situ variable-temperature XRPD (Figure 6). Figure 6a conclusively shows that there are no significant changes in the XRPD pattern of raw taxifolin during heating. Except for the peak at 2*θ* 15.3°, the new patterns of this sample are almost identical to the patterns shown in Figure 6a. Hence, it may be considered that this taxifolin modification is the most thermodynamically stable form. Conversely, phase transitions from taxifolin microtubes to raw taxifolin are evident from the appearance of new peaks during heating (Figure 6b). The transition is observed between 25 and 130 °C (with the appearance of the new peaks at 2*θ* 10.9, 12.1, 18.2, 23.4, 24.8, 28.4, 29.8, 30.9, and 32.6 and disappearance of peaks at 2*θ* 11.6, 15.3, 23.0, 24.0, and 25.9°). Figure 6c visualizes the transformation of the amorphous halo into the patterns of raw taxifolin. Thus, results of XRPD and thermal analysis correlate well with each other.

The objective of this research was to investigate the nature of the different solid states of taxifolin. Our results indicate that taxifolin microspheres are an amorphous substance. This conclusion was confirmed by the presence of an exothermic effect in the thermogram and by an amorphous halo in the XRPD pattern. This form can thus be considered as a polymorphic modification of raw taxifolin according to the definition of polymorphism as defined by the U.S. FDA. At the same time, we found that taxifolin microtubes represent a pseudopolymorphic modification of raw taxifolin. Although XRPD patterns of these forms look quite similar at room temperature, thermal analysis revealed phase transitions between taxifolin modifications. Only the use of comprehensive analysis allowed us to observe phase transitions from a hydrate to an anhydrous form. In this way, the puzzle was put together and we confirmed the pseudopolymorphism of taxifolin. 

Nowadays, the optimization of the API phase state has come to be seen as a new stage of drug development. For this reason, we need to have a clear analysis strategy for such objects. There is a wide range of methods that lend themselves in this respect, however all of them have a combination of advantages and disadvantages [43]. A sole approach might provide weak evidence about the nature of a solid state, and there are several articles reporting inconclusive results from research on different polymorphic forms when only one method was applied [44,45]. Our results concur with those found in previous studies. Integration of substantially different physicochemical analytical methods of analysis gives a chance to avoid ambiguities during drug development. 

Overall, while the nature of taxifolin microtubes and taxifolin microspheres is still being explored, these modifications have the potential to be registered as new APIs. Thermal and microscopic analyses may be used in taxifolin quality control to identify the form of its solid state. Thanks to their morphology, taxifolin microtubes and taxifolin microspheres are promising objects for medical application due to expected unique physicochemical properties [46,47,48].

## 3. Materials and Methods 

### 3.1. Materials

Solid pharmaceutical-grade 2*R*,3*R*-taxifolin (Ametis JSC, Blagoveshchensk, Russia), throughout the paper referred to as raw taxifolin, and urea (99.6%, Carl Roth GmbH, Karlsruhe, Germany) were used in this study. Denatured ethanol (99.8%, Carl Roth GmbH, Karlsruhe, Germany) was used as a solvent.

### 3.2. Preparation of Microtubes

Stock solution was obtained by mixing 1 g taxifolin with urea under 1:1 molar ratio. The mixture was dissolved in 50 mL of denatured ethanol, and deionized water was added dropwise to the stock solution. Liquid samples were stored at room temperature for 48 h, and then the solid phase was isolated from the surfactant via filtration. The precipitate was left to dry in the air for 24 h.

### 3.3. Preparation of Microspheres

The stock solution was prepared by dissolution of 1 kg taxifolin in 40 L of deionized water at 60 °C with continuous stirring. Microspheres were obtained by using GLP-60 centrifugal spray with a blade high-speed disk under the following conditions: inlet air temperature was 180 °C, the outlet temperature was 80 °C. Taxifolin remained in the chamber of the dryer for 1.5–2 s.

### 3.4. SEM

SEM was carried out on JSM-6380LA (JEOL Technics LTD, Akishima, Japan) and involved various magnifications. It was operated at 20 kV accelerating voltage in SEI-mode (Secondary Electron Imaging). Each sample was fixed on an aluminum sample holder with double-sided carbon tape and coated with gold in Argon atmosphere at 0.1 Torr in an IB-3 ion coater (Eiko Engineering Co., Tokyo, Japan). The gold coating was approximately 20 nm thick. As the maximum magnification during SEM analysis was 10,000×, gold layer thickness influence was negligible.

### 3.5. Particle Size Analysis

A laser particle sizer Analysette 22 (Fritsch GmbH, Idar-Oberstein, Germany) was employed to measure the particle size distribution of taxifolin solids. A small aliquot of each powder was dispersed in water. The measuring range was from 0.1 to 1250 µm. For calculations of particle size, we used Fritsch Analysette software.

### 3.6. UV Spectroscopy

The UV spectra were obtained with a Cary 100 spectrophotometer (Varian, Palo Alto, CA, USA). To perform the UV spectroscopy, each taxifolin sample was dissolved in denatured ethanol.

### 3.7. Mass Spectrometry

The mass spectrometry was carried out using an Advion LC-MS mass spectrometer (Advion, Ithaca, NY, USA) equipped with an electrospray ionization source operated in negative ion mode. Each taxifolin sample was dissolved in solvent methanol/water (4:1) with 0.1% of formic acid. Data were collected over a mass range of *m*/*z* 10–1000.

### 3.8. NMR ^1^H

NMR ^1^H spectra of taxifolin samples were recorded on a Varian VNMRS-400 spectrometer (Agilent, Santa Clara, CA, USA) operated at 399.82 MHz at 25 °C in DMSO-*d_6_* in 5 mm sample tubes. The chemical shift was externally referenced to tetramethylsilane.

### 3.9. Thermal Analysis

Specimens with the weights of 2.00–10.00 mg (analytical balance A&D GH-202) were tested with a DSC 204 F1 Phoenix^®^ differential scanning calorimeter, TG 209 F1 Iris^®^ thermobalance, and STA 409 PC Luxx^®^ simultaneous thermal analyzer coupled with QMS 403C Aëolos^®^ quadrupole mass spectrometer (NETZSCH, Selb, Germany). Measurements were taken in aluminum (DSC) and alumina (TG, STA-MS) crucibles (lid with a hole) under dry nitrogen flow (20–70 mL·min^−1^) with a heating rate of 10 °C·min^−1^. All instruments were previously calibrated for temperatures and enthalpies of phase transitions of pure (99.999%) standard substances in compliance with ASTM Practices E967, E968, E1582, and E2253: cyclohexane, Hg, Ga, benzoic acid, In, Sn, Bi, Pb, Zn, CsCl—for DSC; In, Sn, Bi, Zn, Al, Ag, Au—for TG and STA. Calcium oxalate monohydrate was used for validation of thermobalances. Mean estimated temperature and mass determination errors were 0.3 °C and 0.2%. Experimental data were processed in NETZSCH Proteus^®^ Software according to ASTM E794, E2550 and ISO 11357-1.

### 3.10. XRPD

An ARL X’TRA X-ray diffractometer (Thermo Electron Corporation, Waltham, MA, USA) with a vertical *θ*–*θ* wide-angle goniometer and a Peltier solid-state detector with monochromatic Cu-Kα radiation (*λ* = 1.54 Å) operated at 25 mA and 45 kV was used. X-ray powder diffraction (XRPD) data were collected at 295 K. The range of the 2*θ* diffraction angle was 5°–50° with a step size of 0.04° and an integration time of 1 s. Each sample was placed similarly inside the plastic sample holder.

### 3.11. Variable-Temperature Powder X-ray Diffraction

Variable-temperature powder X-ray diffraction measurements were carried out with an ARL X’TRA diffractometer (Thermo Fisher Scientific, Waltham, MA, USA) and HTK2000 (Anton Paar GmbH, Graz, Austria) using Cu-Kα radiation (λ = 1.54 Å) operated at 40 mA and 45 kV. A tungsten block acted as a sample holder. The thickness layer measured ca. 50 μm. Patterns were collected in the 2*θ* range of 10–35° with a step size of 0.05° and 4.0 s counting per step. The copper block with the sample was heated at the rate of 300 °C/min. Temperature control was performed by Eurotherm 2604 (Eurotherm Ltd., Worthing, UK) with BP5\20 thermal element. Data recollection lasted 7 min. Diffraction data were collected at 25, 130, and 170 °C so that changes in structure during heating could be observed. The samples were vacuumed, the residual pressure ranged from 5 × 10^−5^ to 5 × 10^−4^ Pa.

## 4. Conclusions

The array of data we obtained makes it possible to complete the puzzle. Herein, taxifolin API and microtubes were demonstrated to be crystal substances. Tubular modification was characterized by the presence of crystal water; hence it corresponds to a hydrate form. The solid phase of taxifolin API did not contain crystal water. As such, these forms represent pseudopolymorphic modifications. At the same time, microspheres are an amorphous substance. In general, according to the FDA guidelines, both forms of taxifolin may be considered polymorphic modifications of the API.

In this paper, we have also highlighted the need for complex analysis while revealing an API’s polymorphism. Only a multifaceted approach to the problem of polymorphism can prevent biases in drug development.

According to our data, this paper is the first to demonstrate the polymorphic nature of differences among physical and physicochemical properties of new taxifolin forms. It may have a significant impact on the development and registration of new taxifolin-based medicines.

## Figures and Tables

**Table 1:** Morphological parameters of different taxifolin forms.

Sample	Shape	*X*_10_^1^, µm	*X*_50_^1^, µm	*X*_90_^1^, µm
Raw taxifolin	irregular agglomerates	2.16	11.73	46.90
Taxifolin microtubes	tubes	4.18	23.80	214.00
Taxifolin microspheres	spheres	2.22	49.00	190.60

^1^*X*_10_, *X*_50_, and *X*_90_ are particle sizes at undersize values of 10%, 50%, and 90%, respectively.

## Supplementary Materials

The following are available online. Figure S1: Deformations of taxifolin microspheres, Figure S2: UV spectra of different taxifolin forms, Figure S3: Mass spectra of different taxifolin forms, Figure S4: NMR ^1^H spectra of different taxifolin forms, Figure S5: Overlaying plot with DSC curves of different taxifolin forms, Figure S6: STA-MS patterns of taxifolin forms, Table S1: Interpretation of the NMR ^1^H spectra.
