# Supplementary material for: Assembling the Puzzle of Taxifolin Polymorphism
Source: Molecules. 2020 Nov 20;25(22):5437. doi: 10.3390/molecules25225437 (PMC7699767; doi:10.3390/molecules25225437)
Supplement: Supplementary file 1 [file molecules-25-05437-s001.pdf]

**Table S1.** Interpretation of the NMR $^{1}H$ spectra

| Hydrogen Position | δ, ppm | Multiplicity |
|---|---|---|
| -H 3 | 4465 | two doublets |
| -H 2 | 4944 | doublet |
| -OH 3 | 5730 | doublet |
| -H 8 | 5827 | doublet |
| -H 6 | 5874 | doublet |
| -H 5′<br>-H 6′ | 6709 | multiplet |
| -H 2′ | 6842 | singlet |
| -OH 3′ | 8961 | singlet |
| -OH 4′ | 9012 | singlet |
| -OH 7 | 10,798 | singlet |
| -OH 5 | 11,872 | singlet |

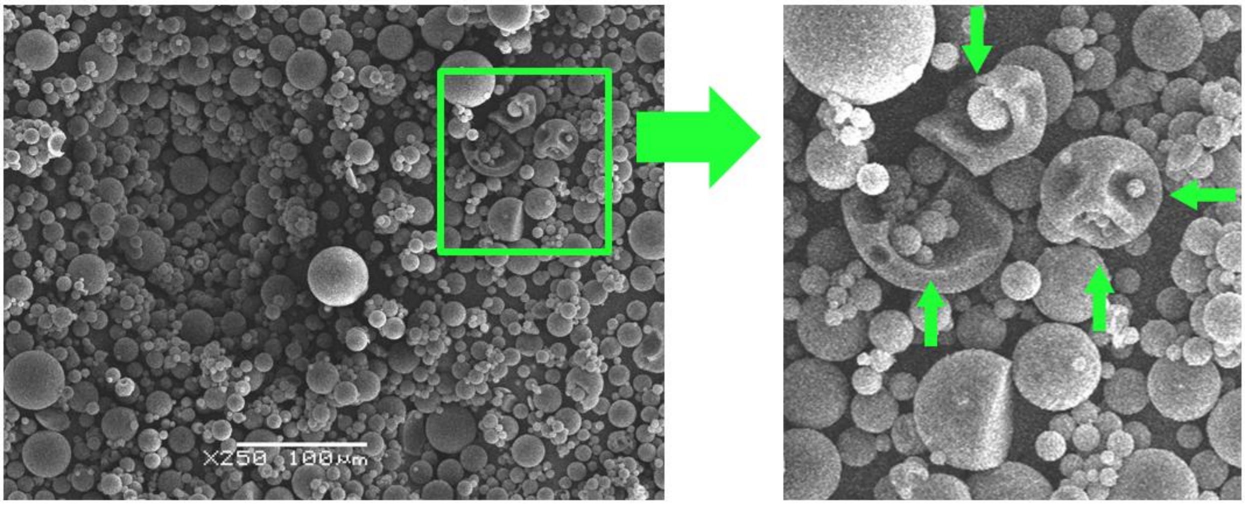


**Figure S1**. Deformations of taxifolin microspheres.

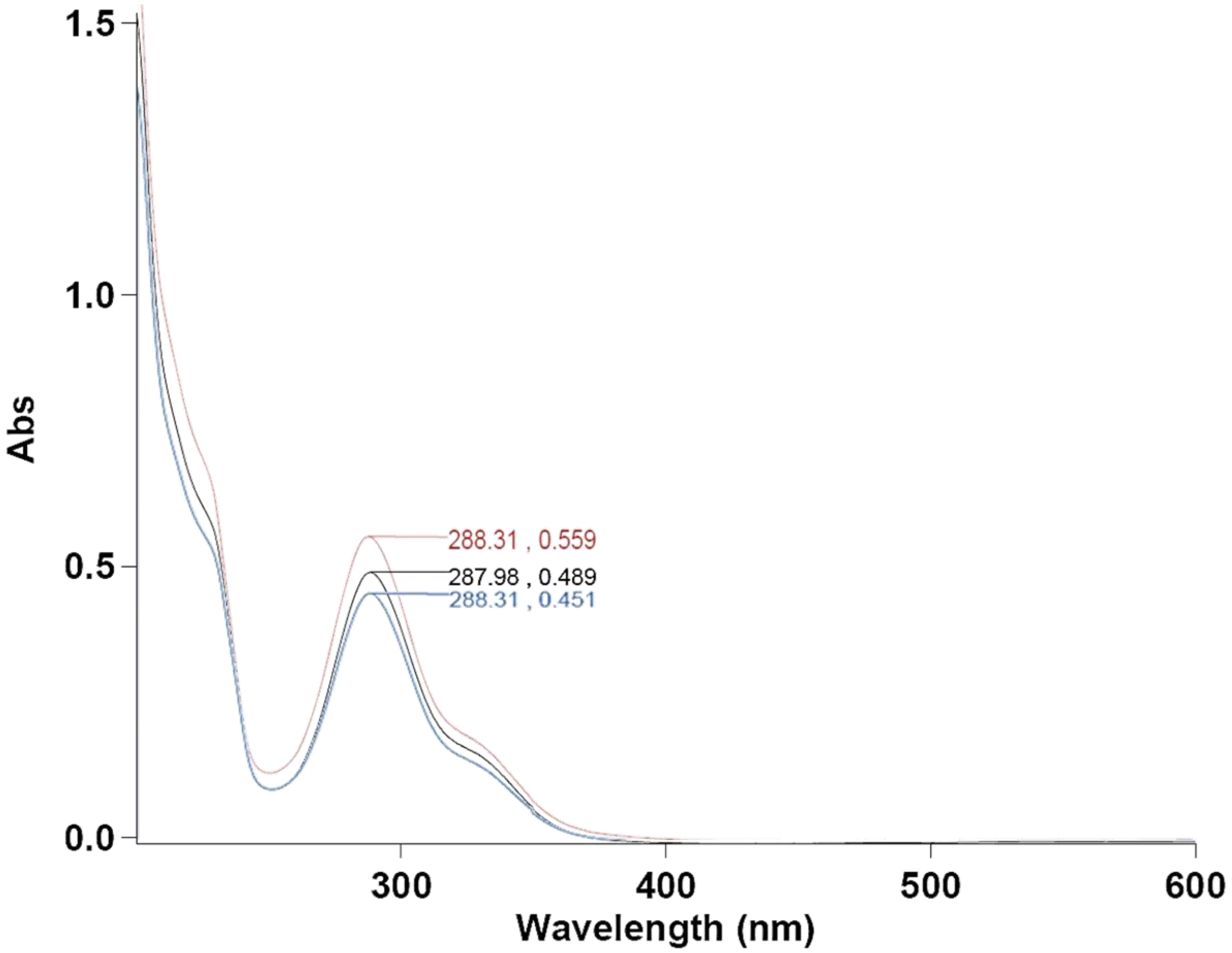


**Figure S2**. UV spectra of different taxifolin forms.

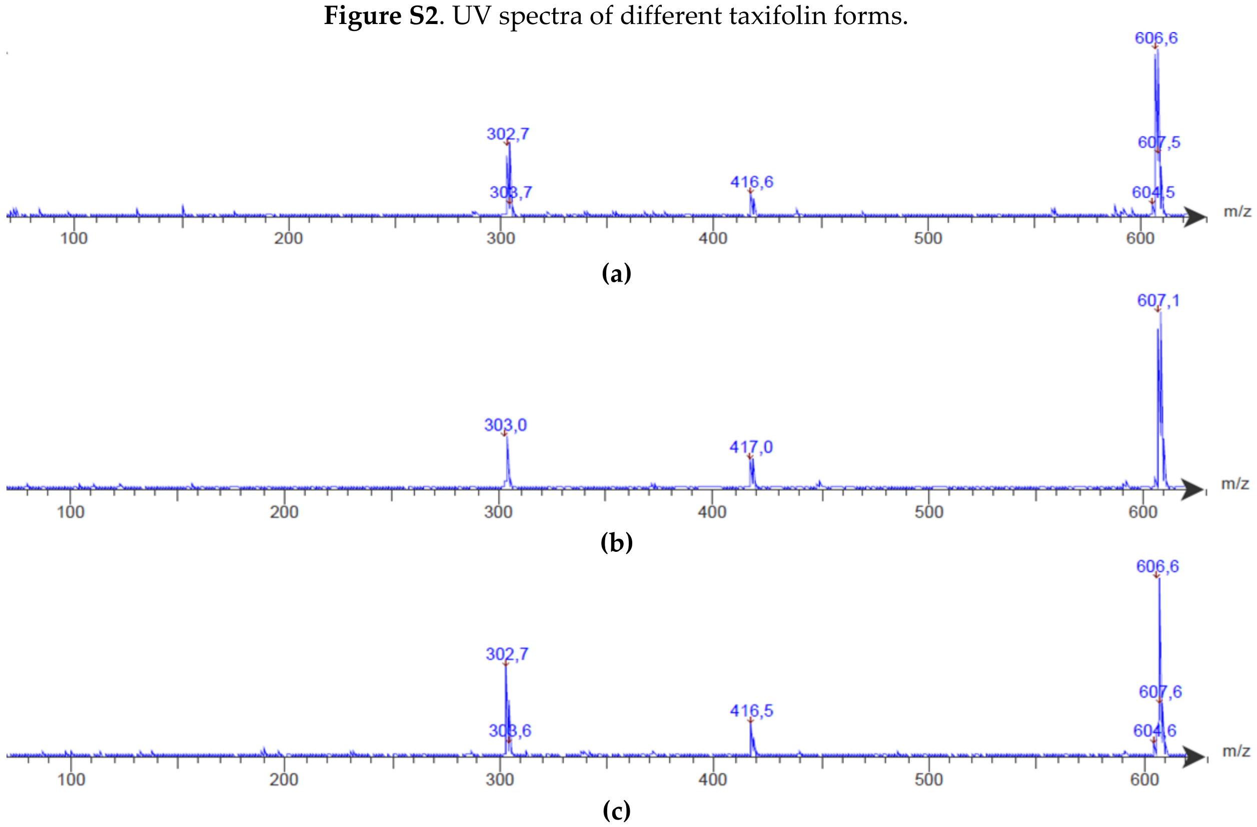


**Figure S3.** Mass spectra of different taxifolin forms.

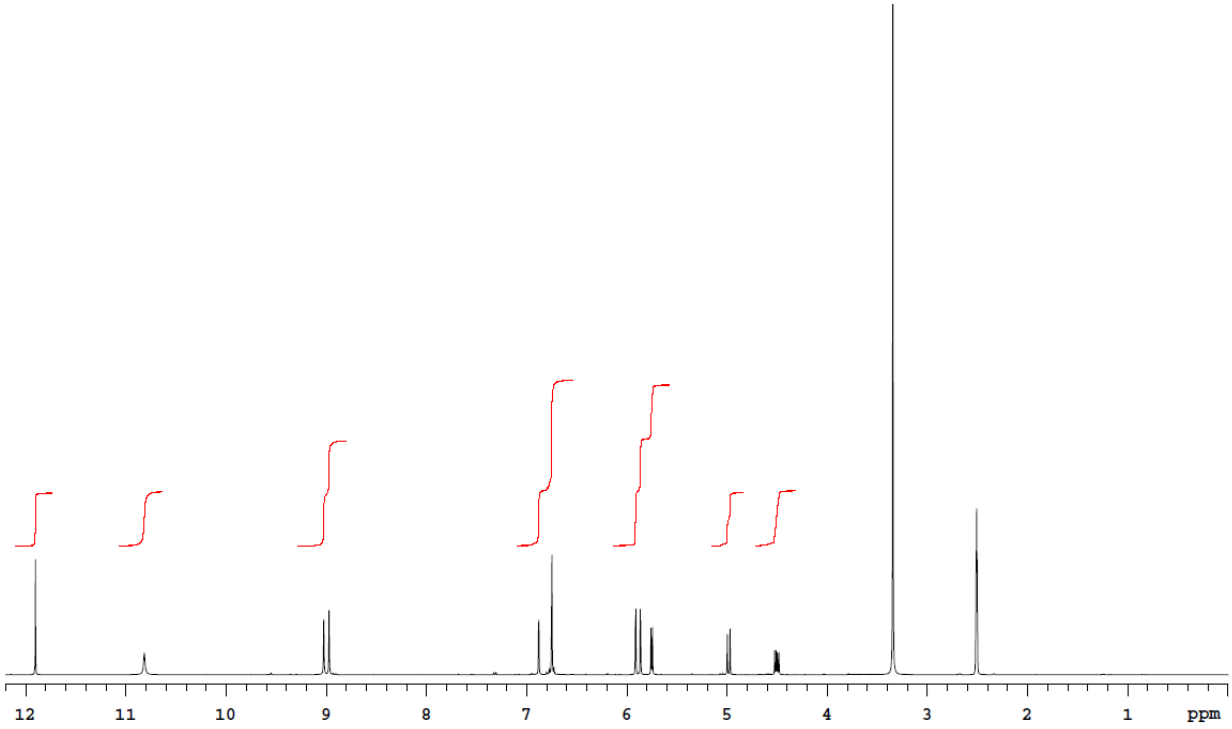


**(a)**

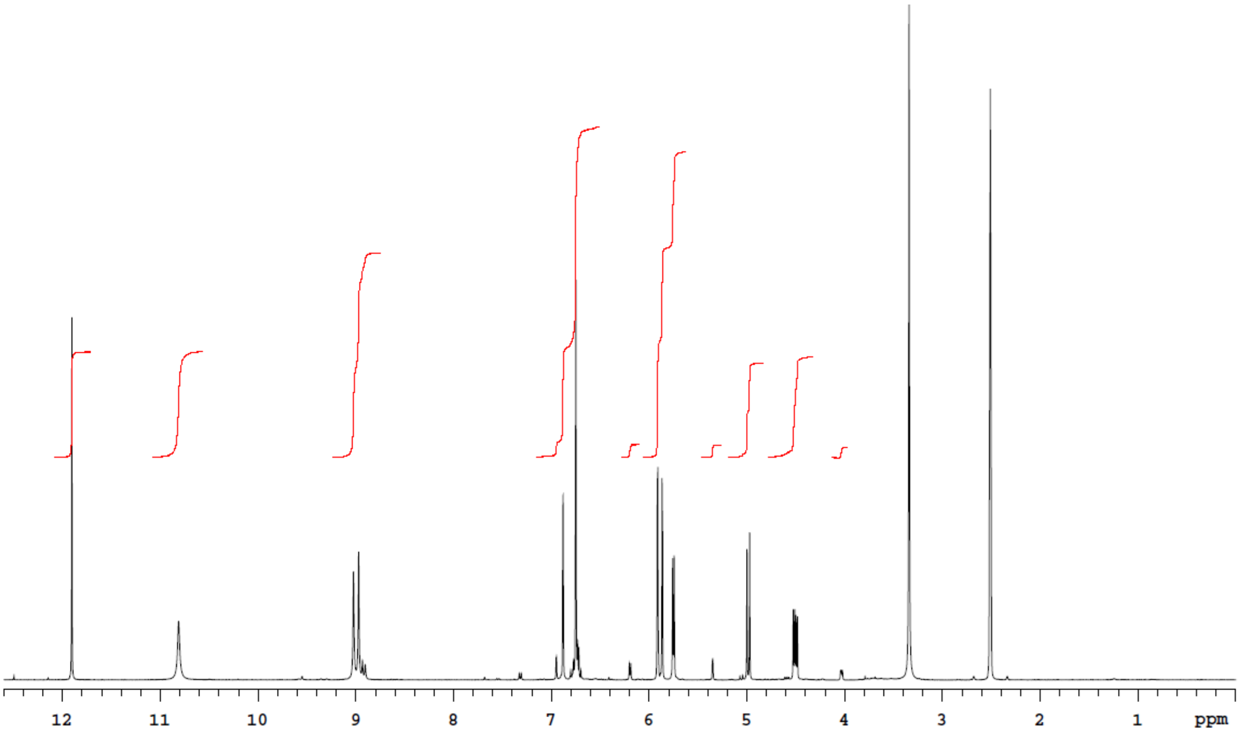


**(b)**

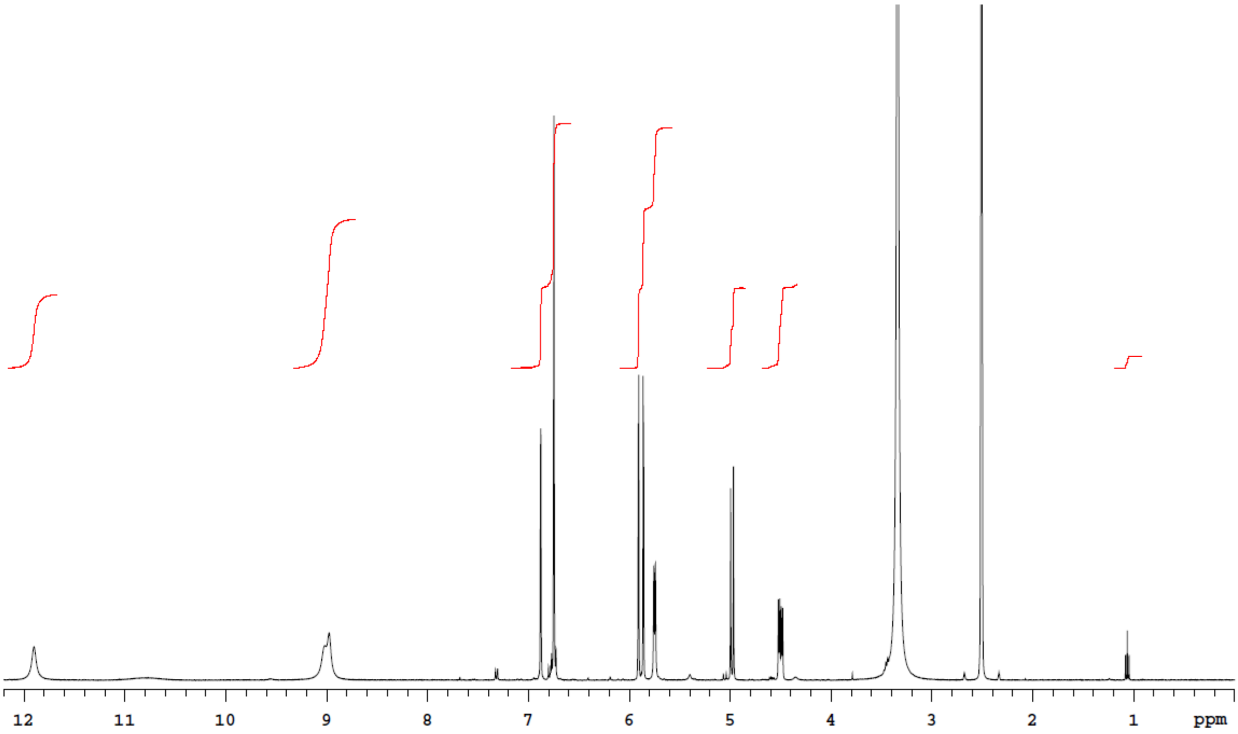


**(c)**

**Figure S4.** NMR ¹H spectra of different taxifolin forms.

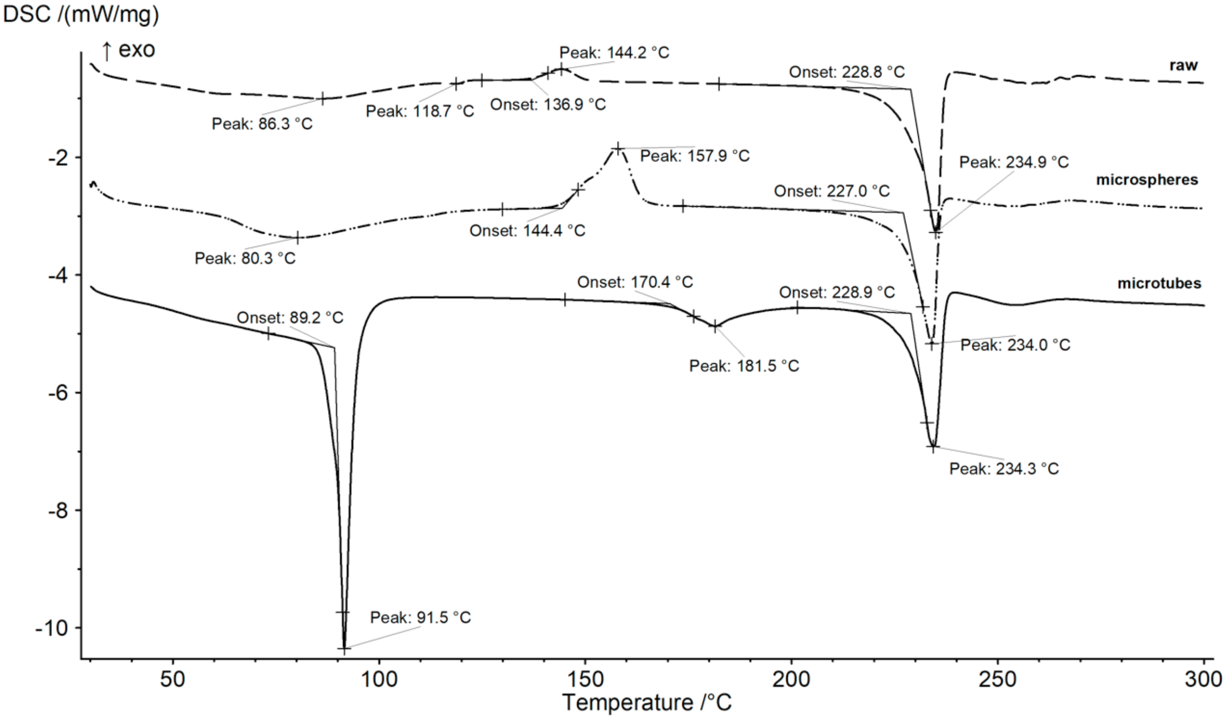


**Figure S5.** Overlaying plot with DSC curves of different taxifolin forms.

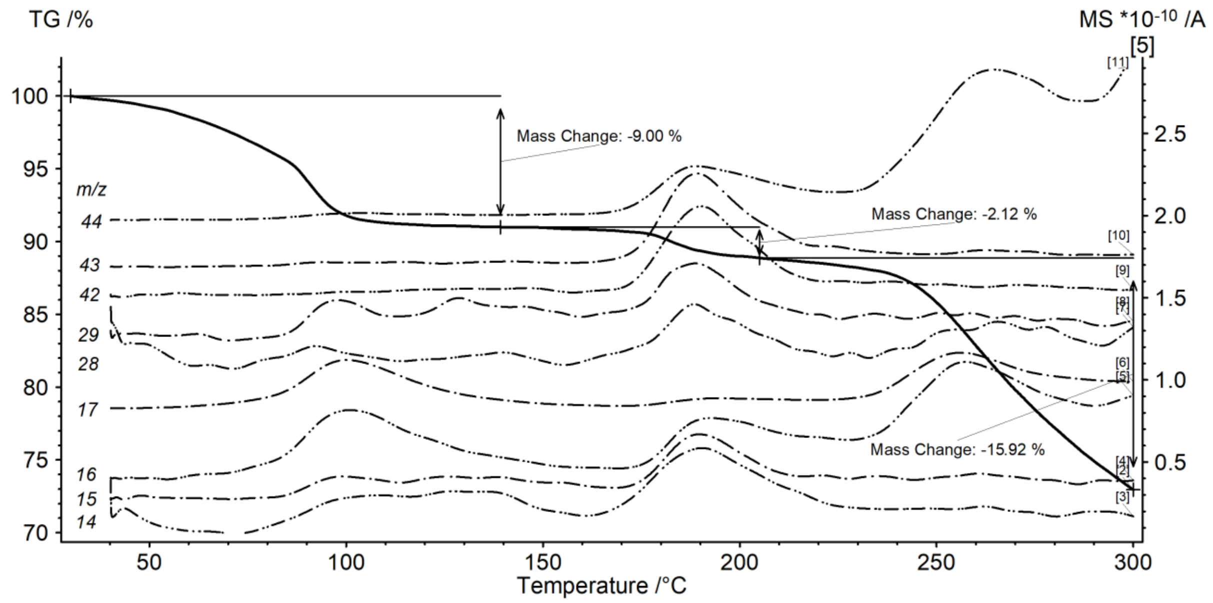


**Figure S6**. STA-MS patterns of taxifolin forms.
